# Research on the Antibacterial Properties of MXene-Based 2D–2D Composite Materials Membrane

**DOI:** 10.3390/nano13142121

**Published:** 2023-07-20

**Authors:** Xiaojie Cheng, Xiaojian Qin, Zhenglun Su, Xun Gou, Zhaomei Yang, Hongshan Wang

**Affiliations:** 1 College of Life Science, Sichuan Normal University, Chengdu 610101, China; qxj965950755@163.com (X.Q.); qq1049435690@163.com (Z.S.); 2 College of Materials and Chemistry & Chemical Engineering, Chengdu University of Technology, Chengdu 610059, China; yangzhaomeimei@163.com (Z.Y.); membranelab323@163.com (H.W.)

**Keywords:** MXene, two-dimensional materials membrane, antibacterial properties, membrane separation, biological pollution

## Abstract

Novel MXene-based two-dimensional (2D) membranes are widely used for water purification due to their highly controllable structure and antibacterial properties. However, in the process of membrane separation, the problems of membrane fouling, especially biological fouling, limits the further application of MXene-based membranes. In this study, in order to improve the antibacterial and separation properties of membranes, three kinds of MXene-based 2D–2D composite membranes (M2~M4) were prepared using polyethersulfone (PES) as the substrate, which were GO@MXene, O-g-C_3_N_4_@MXene and BiOCl@MXene composite membranes respectively. The results showed that the antibacterial activity of M2~M4 against *Escherichia coli* and *Staphylococcus aureus* was further improved, especially the antibacterial ratio of M4 against *Escherichia coli* and *Staphylococcus aureus* was up to 50% and 82.4%, respectively. By comparing the surface morphology of MXene membrane and modified membrane treated bacteria through scanning electron microscopy (SEM), it was found that the cell density on modified membrane was significantly lower than that of pure MXene membrane.

## 1. Introduction

Membrane separation technology is considered to be the most promising separation technology in the 21st century due to its characteristics of environmental protection, low consumption and continuous operation [1,2,3,4,5]. However, during the process of membrane separation in water treatment, membrane fouling emerges as a significant challenge encountered by membranes, with particular emphasis on biological pollution [6,7,8]. Microorganisms will adhere to and grow on the surface of the membranes and secrete extracellular polymers to form biofilms. Aging of the biofilms will decompose and produce proteins, polysaccharides and other substances to promote the adsorption of other microorganisms, causing pollutants to deposit on the surface of the membranes and clog the membrane pores. This leads to a decrease in the separation and permeation performance of the membrane [9,10]. Developing antibacterial membrane materials that possess high permeability, selectivity and stability holds immense importance in advancing membrane water treatment technology [11,12,13,14].

In recent years, two-dimensional materials represented by graphite oxide (GO), metal organic framework (MOF), molybdenum disulfide (MoS_2_), graphite phase carbon nitride (g-C_3_N_4_) and MXenes have demonstrated remarkable accomplishments in the development of high-performance membrane materials, owing to their exceptional physical and chemical properties, as well as their distinctive mass transfer pathways [15,16,17,18,19,20]. Among them, MXene (Ti_3_C_2_T_x_) is a new type of 2D metal carbide or carbonitride, which is popular among 2D membrane separation materials due to its high aspect ratio, biocompatibility, hydrophilicity and good antibacterial properties. It has attracted extensive attention from scholars in the construction and design of antibacterial membranes [21,22,23]. In recent years, the design and regulation of MXene-based membranes have become one of the new ideas in the development of membrane materials. Pandey et al. [24] reported findings on MXene membranes modified with Ag nanoparticles. The addition of Ag shortened the water transport path and increased the permeability from 118 to 420 L·m^−2^·h^−1^·bar^−1^. In addition, the inhibitory effect of the membrane on *Escherichia coli* exceeded 99%. Combining MXene with other two-dimensional materials to construct a 2D–2D composite membrane can overcome the limitations of MXene itself, making the prepared MXene substrate membrane highly permeable and selective, while also improving the stability of the MXene substrate membrane [25,26,27,28,29]. Zhao et al. [30] combined MXene with ZIF-67 to prepare a high-performance gas separation membrane, which showed good chemical stability (CO_2_ and H_2_O resistance), thermal stability and high selectivity for He (selectivity for He/N_2_ and He/CH_4_ gas mixtures is higher than 13).

At present, two-dimensional membranes based on MXene materials have found extensive applications in various fields, including gas separation, water purification and organic solvent separation [31,32,33,34]. However, there is little systematic research on the antibacterial performance of MXene-based two-dimensional membranes. Graphene oxide (GO) has gained significant attention due to its abundant oxygen groups, large specific surface area and excellent adsorption ability. GO-based nanocomposites have found successful applications in various fields, including batteries [35], electronics [36], drug delivery [37], biomedicine [38] and more. Recent studies have focused on combining GO with two-dimensional materials [39] or metal nanoparticles [40] to enhance the antibacterial properties of the composites. O-g-C_3_N_4_ is considered a promising photocatalyst for addressing water pollution issues due to its chemical stability and favorable band gap [41]. BiOCl, another photocatalyst, is primarily used for water pollution treatment [42]. While scientists have made efforts to improve the light absorption range and reduce the electron–hole recombination rate of BiOCl, there is limited research comparing the antibacterial performance of these two photocatalysts. Therefore, in this work, MXene was combined with two-dimensional materials GO, oxygen-doped graphite phase carbon nitride (O-g-C_3_N_4_) and bismuth oxychloride (BiOCl) to construct three kinds of MXene-based 2D–2D composite membranes using vacuum-assisted self-assembly. The separation ability of the three 2D–2D modified membranes and the pure MXene membrane to dyes and the bacteriostasis to *S. aureus* and *E. coli* were systematically studied and compared. This study is expected to establish a certain reference basis for the development of new MXene based 2D–2D antibacterial membrane materials to alleviate the problem of biological pollution.

## 2. Materials and Methods

### 2.1. Materials

MAX (Ti_3_AlC_2_) powder was purchased from China Jilin 11 Technology Co., Ltd. (Jilin, China). PES membrane was produced by Tianjin Jinteng Experimental Equipment Co., Ltd. (Tianjin, China). Sulfuric acid (H_2_SO_4_), sodium nitrate (NaNO_3_), phosphoric acid (H_3_PO_4_), potassium permanganate (KMnO_4_), anhydrous copper sulfate (CuSO_4_), ammonium oxalate ((NH_4_)_2_C_2_O_4_·H_2_O), urea (CH_4_N_2_O) and hydrochloric acid (HCl) were bought from Chengdu Kolong Co., Ltd. (Chengdu, China). Bi(NO_3_)_3_⋅5H_2_O, ammonium persulfate (H_8_N_2_O_8_S_2_), Congo red (CR) and lithium fluoride (LiF, 99%) were provided by Solarbio Life Sciences (Beijing, China).

### 2.2. Synthesis of Two-Dimensional Nanosheets

MXene (Ti_3_C_2_T_x_) materials were prepared by chemical etching of the MAX (Ti_3_AlC_2_) phase [43,44]. Firstly, a beaker containing 20 mL of 9 M HCl solution was used to slowly mix 0.5 g of LiF, followed by continuous stirring for 30 min. Then, 0.5 g MAX phase was added to the mixed solution and the temperature was maintained at 30 °C with magnetic stirring for 20 h. After that, the dispersion obtained was centrifuged repeatedly (3500 rpm, 10 min) using a centrifuge and washed repeatedly with deionized (DI) water until pH < 6. Multilayer MXene nanosheets were obtained by collecting the supernatant, and the samples were dissolved in 200 mL of DI water and sonicated at room temperature (25 °C) under nitrogen atmosphere for 6 h. The suspension obtained through sonication underwent subsequent centrifugation at a speed of 8000 rpm for a duration of 30 min. The resulting supernatant was collected and subjected to a final freeze-drying process, yielding two-dimensional MXene nanosheets.

### 2.3. Preparation of Other Two-Dimensional Materials

In this work, GO was prepared by an improved Hummers method [45]. Firstly, the graphite powder was dissolved in a beaker containing NaNO_3_ and H_2_SO_4_ solutions, and stirred continuously for 1.5 h to obtain pre-graphite oxide. Then, KMnO_4_ was slowly added to the beaker for 1 h to further oxidize it. After continuous stirring for 2 h, DI water and hydrogen peroxide (H_2_O_2_) solution were added to the beaker successively to remove the unreacted KMnO_4_. The graphene oxide (GO) was subjected to multiple DI water washes in order to achieve neutralization of remaining acid until a neutral solution was obtained. Finally, the GO was stripped ultrasonically and the synthesized GO nanosheets were collected, after which the GO powder was obtained by freeze-drying.

O-g-C_3_N_4_ was synthesized via thermal polymerization [46]. In short, 10 g urea powder was firstly added to a beaker containing DI water to obtain urea suspension, and then 1 g ammonium oxalate powder was added to the suspension, which was then placed on a magnetic agitator and magnetically stirred for 1 h at room temperature. The solution was then dried in a vacuum drying oven (80 °C) until the water in the solution completely evaporated, resulting in a white powder. The white powder was then placed in a crucible and heated for 2 h at a rate of 5 K·min^−1^. When the crucible was cooled, the crucible was removed and the resulting yellow powder was collected, namely O-g-C_3_N_4_.

BiOCl was prepared via a low-temperature chemical process [47]. The specific experimental operation was as follows: Firstly, 4.85 g Bi(NO_3_)_3_·5H_2_O was dissolved in a beaker containing 20 mL HNO_3_, denoting it as solution A. Then, 5.96 g KCl was dissolved in 100 mL DI water and recorded as solution B. Solution A was subjected to continuous stirring at a temperature of 30 °C, while solution B was gradually introduced into solution A through dropwise addition, ensuring simultaneous stirring. After continuous stirring for 30 min, the samples underwent separate washes with ethanol and DI water. The sample was subsequently subjected to a drying process in a blast drying oven at 60 °C for 12 h, then the white powder was obtained as BiOCl.

### 2.4. Preparation of Two-Dimensional Composite Membranes

**Figure 1:**
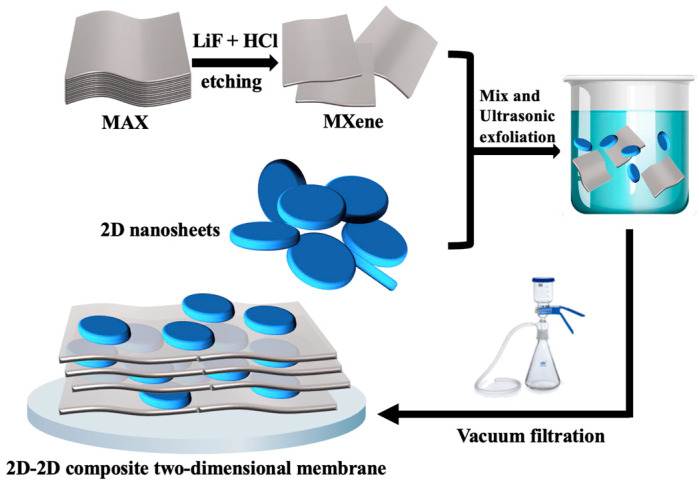
Preparation of MXene-based 2D–2D composite two-dimensional membrane.

The synthesis scheme for the preparation process of MXene, GO@MXene, O-g-C_3_N_4_@MXene and BiOCl@MXene composite two-dimensional membranes is shown in Figure 1. Specifically, a certain amount of MXene and other 2D materials (GO, O-g-C_3_N_4_ and BiOCl) were individually dispersed in 50 mL of DI water within separate beakers, and ultrasonic treatment was conducted at room temperature for a duration of 25 min. Then, the MXene dispersion was mixed with other 2D material dispersions and continued to be ultrasonically treated at room temperature for 15 min to obtain uniform GO@MXene, O-g-C_3_N_4_@MXene and BiOCl@MXene precursor solutions, respectively. Finally, the precursor solutions were vacuum filtered onto the PES substrate to prepare different MXene-based 2D–2D composite membranes. Table 1 shows the mass ratio of materials in all composite membranes. The MXene membrane, GO@MXene membrane, O-g-C_3_N_4_@MXene membrane and BiOCl@MXene membrane were labeled as M1~M4, respectively.

### 2.5. Characterization

The crystal structures of MAX, MXene, GO, O-g-C_3_N_4_ and BiOCl were characterized using an X-ray diffractometer (XRD; D8advance, Billerica, MA, USA). Characteristic peaks of the samples were detected using Fourier transform infrared spectroscopy (FTIR, Nicolet iS50, Waltham, MA, USA). The morphology of materials, the surface morphology and cross-sectional morphology of the two-dimensional membranes and the bacterial density on the surface of the original membrane and modified membrane were analyzed using scanning electron microscopy (SEM, JSM-7500F, JEOL, Japan). The absorbance of the dye solution before and after the test was recorded with a UV–Vis spectrophotometer (UV756CRT, Shanghai, China).

### 2.6. Permeability and Selectivity of Membranes

The permeability of the membranes was assessed by quantifying the flux of pure water with a volume of 100 mL through the vacuum filtration device. To obtain a constant flux of pure water, the membrane was pre-pressurized for more than 10 min before recording the flux. The permeability (*P*) can be determined using Equation (1), where *V* denotes the volume of pure water (L), *A* represents the effective membrane area (m^2^), *t* signifies the permeation time (h) and ∆p corresponds to the permeation pressure (bar).

(1) $$P=\frac{V}{A\times t\times ∆p}$$

The separation performance of composite membranes was measured using CR (100 ppm) as a typical pollutant, and the concentration of CR before and after separation was measured utilizing a UV spectrophotometer to obtain the retention rate (*R*). (*R*) can be calculated according to the following equation:

(2) $$R(\%)=\left(1-\frac{C_{p}}{C_{f}}\right)\times 100\%$$

where $C_{f}$ represents original concentration (g/L) and $C_{p}$ represents filtrate concentration (g/L).

### 2.7. Antibacterial Test

The antibacterial activity of the synthesized samples (MXene, GO@MXene, O-g-C_3_N_4_@MXene, BiOCl@MXene) was evaluated against two bacterial strains: *Staphylococcus aureus* (Gram-positive) and *Escherichia coli* (Gram-negative). Both turbidimetric and zone of inhibition methods were employed to assess the antibacterial properties of the membranes. In brief, the experimental bacterial strain was cultured in sterile Luria–Bertani (LB) broth at 200 rpm and 37 °C for 8 h. Subsequently, the culture was inoculated into 50 mL of fresh LB medium containing the different synthesized samples. Samples were collected every two hours to measure their optical density (*λ* = 600 nm) over a period of 24 h. Simultaneously, membrane samples of approximately 1.57 cm^2^ were immersed in LB medium overnight. Afterward, the suspensions collected at different time points were transferred onto LB agar plates and incubated for 16 h. The agar plates were used to count the colony-forming units (CFU), enabling the quantification of the antibacterial effectiveness of the membranes at a macroscopic scale. The viability of each sample was calculated using:

(3) $$survival=\frac{Treatment\;colony}{Control\;colony}\times 100\%$$

The method for obtaining SEM images was further optimized [48]. A bacterial suspension of 500 µL with a concentration of 1 × 10^6^ CFU was injected into LB solid medium. The composite membrane (~1.57 cm^2^) was then placed on the solidified plate for 12 h and incubated at 37 °C. After cultivation, the sample was removed from the Petri dish and the surface of the membrane was rinsed with sterile phosphate buffer (pH 7.4) to remove any unattached bacteria. To fix the bacteria on the membrane surface, 2.5% glutaraldehyde was applied for 24 h at 4 °C. Subsequently, the membranes were washed with 0.1 M phosphate buffer (pH 7.4) and dehydrated using a graded series of ethanol concentrations (30%, 50%, 70%, 90% and 100%). The samples were subsequently subjected to a thorough vacuum-drying process at 40 °C for 2 h and observed under a scanning electron microscope (SEM) after sputter-coating with gold.

## 3. Results

### 3.1. Characterizations of Material

**Figure 2:**
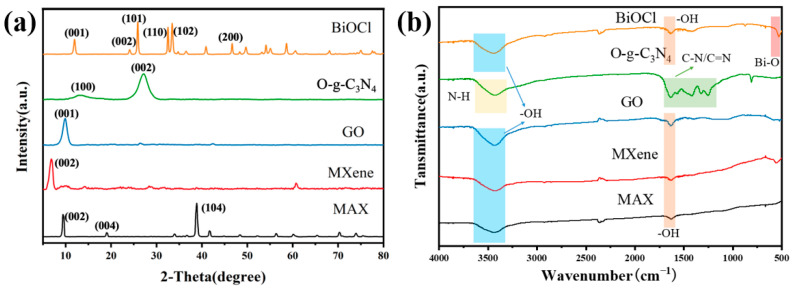
(**a**) XRD and (**b**) FTIR spectrums of MAX, MXene, GO, O-g-C_3_N_4_ and BiOCl.

MXene, GO, O-g-C_3_N_4_ and BiOCl were characterized by XRD. As shown Figure 2a, only the characteristic peak in the (002) plane was present for MXene, where the complete disappearance of the characteristic peak in the (104) plane belonging to Al proves the successful preparation of MXene [49]. In addition, there was a characteristic peak in the (001) plane in the spectrum of GO. The peaks at 13° and 27° in the spectrum of O-g-C_3_N_4_ belonged to the (100) and (002) planes, respectively [50]. In addition, according to the spectra of BiOCl, the peaks at 12.0°, 24.0°, 25.6°, 32.3°, 33.4° and 46.7° belonged to the (001), (002), (101), (110), (102) and (200) planes of BiOCl, respectively [51]. The diffraction peaks of the aforementioned 2D materials were all very similar to those in the literature [46,47,49,50,51]. Figure 2b depicts the FTIR spectra of different two-dimensional nanomaterials. The vibrational frequency of 537.6 cm^−1^ was specifically associated with the Bi-O bond present in BiOCl [52]. In the spectrum of O-g-C_3_N_4_, the peaks at 1800–900 cm^−1^ were typical stretching vibration modes of C=N and C-N heterocycles, while the peak at 3400 cm^−1^ was caused by N-H stretching [53,54,55,56]. The peaks appearing at 1619.8 and 3425.8 cm^−1^ in the spectra were due to the presence of hydroxyl groups on the surfaces of MAX, MXene, GO and BiOCl [57]. These above results indicate that MXene, GO, O-g-C_3_N_4_ and BiOCl were successfully prepared.

**Figure 3:**
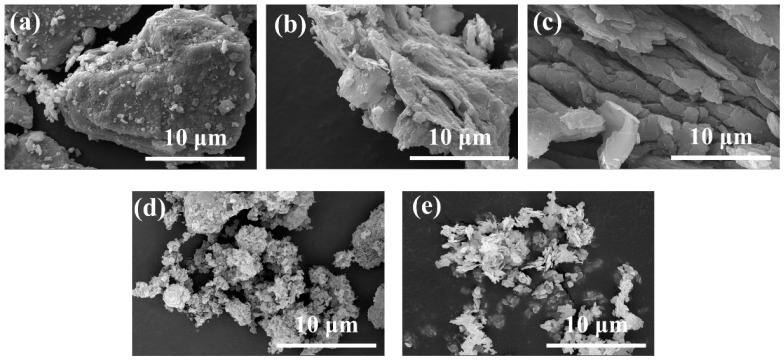
The SEM images for (**a**) MAX, (**b**) MXene, (**c**) GO, (**d**) O-g-C_3_N_4_ and (**e**) BiOCl.

The scanning electron microscopy (SEM) technique was utilized to characterize the surface morphology of the two-dimensional material. In Figure 3a,b, compared with the dense lamellar MAX phase, the MXene powder with the aluminum layer etched off had a loose structure and appeared to be layered. The surface morphology of GO was similar to that of MXene, both showing a lamellar structure (Figure 3c) [58]. In addition, the O-g-C_3_N_4_ and BiOCl particles were both uneven in size but also had a distinct lamellar structure (Figure 3d,e) [47,59].

### 3.2. Characterizations of Membranes

**Figure 4:**
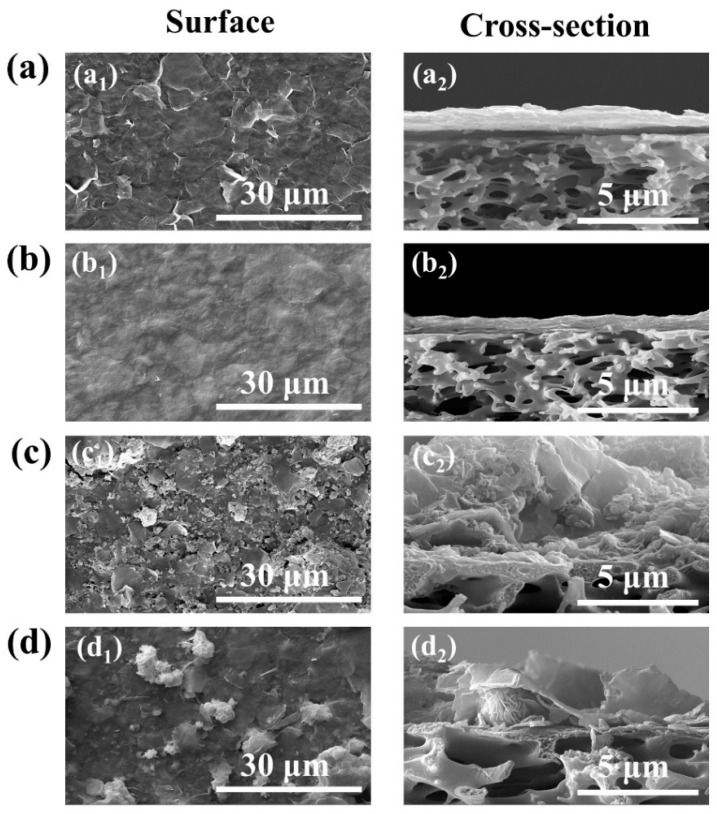
(**a**–**d**) The surface and cross-section SEM images for M1~M4.

Figure 4 presents the surface and cross-sectional scanning electron microscopy (SEM) images of membranes M1 to M4. As shown in Figure 4(a_1_,b_1_), M1 and M2 exhibited typical 2D membrane structures with both smooth and flat surfaces. The thickness of M1 was about 1 μm (Figure 4(a_2_)), whereas the thickness of the M2 separation layer was about 0.6 μm (Figure 4(b_2_)). Two similar 2D materials (MXene and GO) were co-blended and stacked more tightly than the pure membrane after vacuum filtration. In addition, the surface of M3 was folded (Figure 4(c_1_)), and the SEM cross-sectional view shows a membrane thickness of about 6.0 μm (Figure 4(c_2_)). In addition, many flower-like nanoparticles were present on the surface of M4 membrane (Figure 4(d_1_)), which was caused by the incorporation of BiOCl. The thickness of the separated layer in M4 membrane was about 3 μm (Figure 4(d_2_)). It can be seen that after the modification of two-dimensional materials O-g-C_3_N_4_ and BiOCl, the thickness of the membrane separation layer of M3 and M4 was significantly higher than that of M1, which can be attributed to the presence of larger-sized particles and irregular distribution of O-g-C_3_N_4_ and BiOCl.

### 3.3. Permeability and Selectivity of Membranes

**Figure 5:**
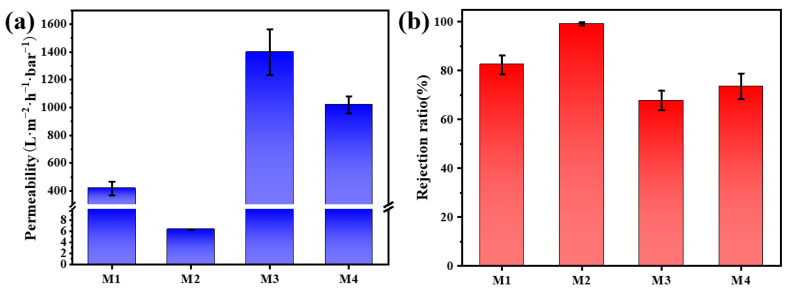
(**a**) The permeability of M1~M4 and (**b**) the rejection ratio of CR by M1~M4.

In this study, the permeability of composite membranes was studied by using a dead-end filtration device to test the pure water permeability. As shown in Figure 5a, under the condition of 0.1 MPa, the permeability of pure MXene membrane was 417.79 L⋅m^−2^⋅h^−1^⋅bar^−1^. After GO modification of MXene, due to the tightly ordered stacking of MXene and GO nanosheets, the interlayer channels of the membrane were reduced, resulting in a significant decrease in the permeation flux of M2 (6.48 L⋅m^−2^⋅h^−1^⋅bar^−1^) [45]. In contrast, the pure water permeability flux of M3 and M4 was significantly increased, up to 1397.37 L⋅m^−2^⋅h^−1^⋅bar^−1^ and 1019.36 L⋅m^−2^⋅h^−1^⋅bar^−1^, respectively. This was due to the interweaving of O-g-C_3_N_4_ and BiOCl nanomaterials with MXene, which formed many new nanochannels in the membrane structure for molecular transport [50,60]. The removal ratios of different composite membranes for simulated wastewater Congo red (CR) dye are shown in Figure 5b, and the removal rates of CR from M1 to M4 were 82.42%, 99.12%, 67.71% and 73.53%, respectively. It is noteworthy that the permeability and the pollutant rejection ratio exhibited an opposite trend, which was consistent with the “trade-off” effect to some extent [61].

### 3.4. Antimicrobial Behavior Test of Membranes

**Figure 6:**
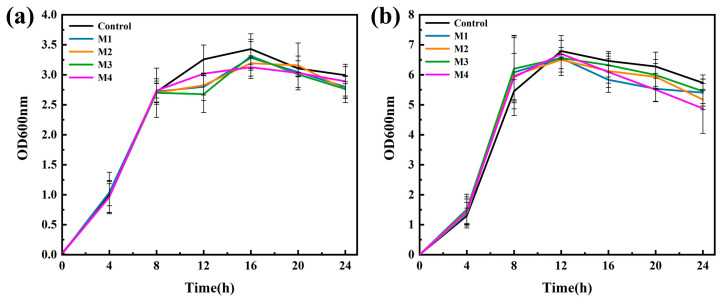
Growth curves of (**a**) *E. coli* and (**b**) *S. aureus* after being treated with different membranes (M1~M4). Control represents the growth curve of bacteria in the absence of membrane material.

The antibacterial effect of several materials on *E. coli* and *S. aureus* was evaluated using a turbidity method. Figure 6 presents the impact of the synthesized samples on bacterial growth using the turbidimetric method. The bacterial strain without any membranes was operated as a control. The results demonstrate that several materials exhibited potent inhibitory effects on pathogenic bacteria. In the case of *E. coli*, the optical density began to decrease after 8 h of treatment with the materials compared to the control group (Figure 6a). Conversely, for *S. aureus*, the decline in optical density was observed after 12 h of treatment (Figure 6b). This can be attributed to the thick and dense peptidoglycan layer present in the cell wall of Gram-positive bacteria, which limits the material’s ability to fully exhibit its bacteriostatic properties.

**Figure 7:**
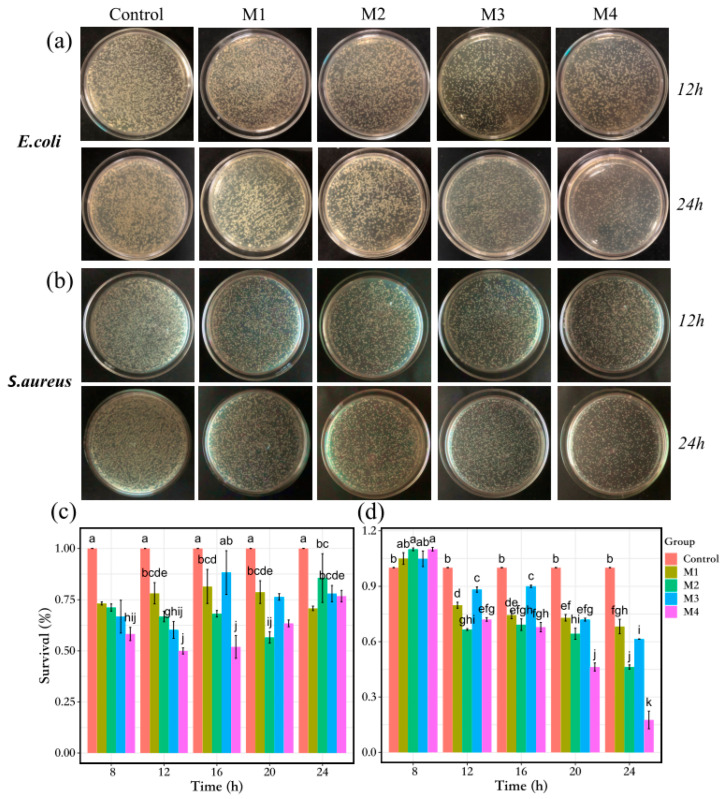
Digital images of (**a**) *E. coli* and (**b**) *S. aureus* colonies after treatment with different membranes (M1~M4), respectively. The relative viability of bacteria after incubation of *E. coli* (**c**) and *S. aureus* (**d**) with different membranes (M1~M4) was determined by plate counting after 24 h. Control represents the growth condition of bacteria in the absence of membrane material (different letters a–k are compact letters display of significant differences).

The antibacterial activity of multiple materials was quantitatively evaluated using a CFU counting method. Figure 7a,c illustrate the bacteriostatic performance of the materials against *E. coli*, which was found to be superior to their effect on *S. aureus*. Pure MXene membrane exhibited a relatively consistent bacteriostatic rate regardless of co-treatment. Among the synthesized samples, M4 demonstrated excellent bacteriostatic performance against *E. coli*, with a 50% inhibition rate at 12 h, twice that of pure MXene membrane. M3 also exhibited good bacteriostatic properties, achieving a rate of 39.7% against *E. coli* at 12 h. On the other hand, M2 took a longer time to exhibit its bacteriostatic effect, reaching 43.4% inhibition at 20 h. When co-cultured with *S. aureus* for 8 h (Figure 7b,d), the materials did not exert antibacterial properties until 12 h of co-treatment, consistent with the growth curve. Several composites exhibited similar bacteriostatic properties at 12 h, 16 h and 20 h, with the antibacterial activity of the membranes peaking at 24 h. M4 showed the highest bacteriostatic rate at 82.4%, followed by M2 at 53.7%, M3 at 38.5% and pure MXene membrane at 31.7%. This may be attributed to *S. aureus* reaching the decay stage at this point, allowing for sufficient interaction between the composite membrane and the bacteria.

**Figure 8:**
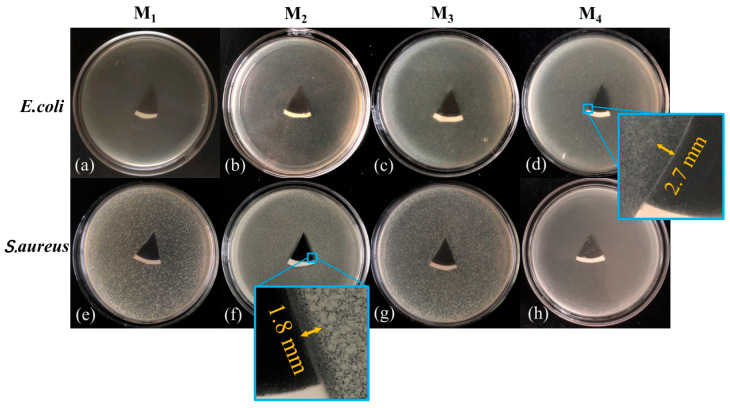
The zone inhibition produced by different membranes (M1~M4) against *S. aureus* (**a**–**d**) and *E. coli* (**e**–**h**).

Furthermore, the four types of membranes were placed on plates containing *E. coli* and *S. aureus* (Figure 8). After 12 h of inverted incubation, it was observed that the width of the antibacterial zone for the plate with *E. coli* and M4 composite membranes reached 2.7 mm, which was significantly larger than the other membranes. In contrast, there was almost no discernible bacteriostatic zone on the plates with M3 and pure MXene membrane. However, the inhibition zone width was greater for M2 compared to M4 on the plate containing *S. aureus*. This observation further indicates that M4 had a stronger inhibitory effect on *E. coli*. As a novel semiconductor catalytic material, BiOCl exhibited inhibitory effects on both *E. coli* and *S. aureus* when combined with M1, even in the absence of light, which aligns with previous studies [60].

**Figure 9:**
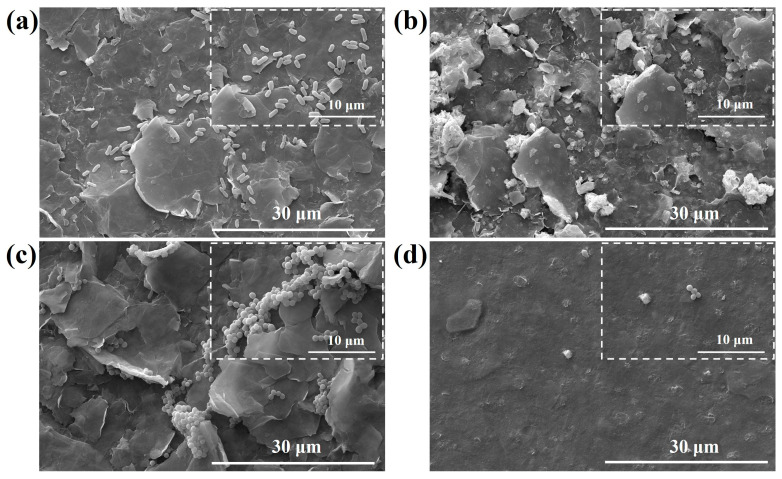
SEM image of the *E. coli* after treatment with M1 (**a**) and M4 (**b**) and the *S. aureus* after treatment with M1 (**c**) and M2 (**d**) (scale bar: 30 μm and 10 μm).

To gain further insights into the interaction between the bacteria and membrane surface, the surface morphologies of M1, M2 and M4 after 12 h of incubation were examined using SEM (Figure 9). The bacterial cells on M2 and M4 exhibited noticeable differences compared to those on the pure MXene membrane. The densities of surviving cells on the M2 and M4 samples were significantly lower than on the pure MXene membrane. To process the samples, any loosely attached bacteria were removed by rinsing with sterile phosphate buffer. Live bacteria tend to adhere to the membrane surface through specific proteins and secrete polysaccharides, enhancing their adhesion to the material surface [62]. In contrast, dead bacteria are more easily washed away. Furthermore, it was observed that the cells on the pure MXene membrane surface appeared smooth and plump, and exhibited no signs of membrane disruption or contraction. They were overlapping with each other on the surface. Figure 9b reveals the presence of rough features and irregular depressions on the *E. coli* cells, along with a reduced number of surviving cells, suggesting a higher level of damage caused by M4. Additionally, the number of *S. aureus* cells on the M2 membrane was significantly lower compared to the pure MXene membrane (M1). The surface of bacterial cells on the M2 membrane appeared rough, with inward wrinkles and cracks, indicating extensive damage and the release of bacterial cell contents. These observations suggest that M2 and M4 achieved a bactericidal effect by damaging the cell membrane. In summary, the SEM surface analysis of bacteria treated with different materials demonstrates that incorporating MXene with various substances significantly enhances its bacteriostatic properties.

The unique layered structure of BiOCl, as a type of semiconductor photocatalyst, has been extensively researched. However, most studies have focused on enhancing the light absorption range and the recombination rate of electron–hole pairs in BiOCl through doping noble metals [42], ions [63] and hybrid heterojunctions [64]. These modifications aim to improve the photocatalytic degradation of water pollution [65,66,67]. The mechanisms underlying bacterial inactivation by BiOCl have received limited attention. Su et al. [42] discovered that the antibacterial effect of Au/BiOCl with different morphologies is significant under visible light irradiation. Fan et al. [68] demonstrated that PPy-BiOCl exhibits effective antibacterial activity of 99.25% and 99.23% against *S. aureus* and *E. coli*, respectively, under near-infrared (NIR) light. This effect is attributed to the generation of ROS and the photothermal effect of PPy-BiOCl, which rapidly and effectively destroy bacterial membranes. Song et al. [69] proposed a molecular mechanism, stating that intracellular ROS formation occurs in *S. aureus* bacteria in the presence of BiOCl-KIT-6, leading to bacterial eradication. In a previous study [60], we found that BiOCl-PPy@MXene also exhibits bacteriostatic properties in the absence of light. However, the antibacterial mechanism of BiOCl as a photocatalytic material on *E. coli* and *S. aureus* in the absence of light has not been extensively investigated. In this study, we observed a certain level of antimicrobial activity. Our findings suggest that bacteria tend to adhere and deposit on the irregular and rough surfaces of materials, providing favorable colonization sites [41]. Su et al. [70] demonstrated that the flower-like Au/BiOCl nanocomposite exhibited the highest photocatalytic bactericidal performance among the prepared samples. Therefore, we speculate that M4 is more likely to adhere to bacteria due to its physical structure, enabling comprehensive interaction with bacteria. To gain further insights into the antibacterial performance, we plan to investigate the interaction between BiOCl and the bacterial cell wall in the absence of light using a three-electrode system [71]. Additionally, in future studies, we aim to determine whether BiOCl can generate reactive oxygen species under dark conditions [72], as these species can damage bacterial cell components and influence bacterial survival. Graphene oxide (GO) is widely employed in the field of antibacterial research due to its unique structure, high biocompatibility and design flexibility. Various strategies have been developed to create composites with antimicrobial properties. Li [73] discovered that the structures of *E. coli* and *S. aureus* cells can be severely damaged, leading to protein leakage, when CSCl@GO composites are introduced. Da [74] investigated the interaction mechanism between GO-Ag sheets and *E. coli* using TEM, revealing that GO-Ag accumulates on the bacterial cell surface, resulting in cell inactivation. Comparatively, M2 demonstrates a better antibacterial effect on *S. aureus* compared to pure MXene membrane. This can be attributed to MXene’s characteristic two-dimensional layered structure, which provides a larger surface area. After incorporating GO, the resulting composite exhibits a larger size, leading to a stronger trapping effect on bacteria. The trapping effect restricts nutrient absorption by bacteria, ultimately leading to their demise [75]. While M3 can inhibit the growth of both *E. coli* and *S. aureus*, its effectiveness is relatively low compared to the other two composite membranes. This may be due to the fact that O-g-C_3_N_4_, being a photocatalytic material, is unable to fully exhibit its optimal bacteriostatic properties in the absence of light.

## 4. Conclusions

In conclusion, three types of MXene-based 2D–2D composite membranes were prepared by combining MXene with GO, O-g-C_3_N_4_ and BiOCl using vacuum-assisted self-assembly. The successful preparation of 2D materials was confirmed through XRD, FTIR and SEM characterization. Compared with the pure MXene membrane (M1), the addition of other 2D materials enhanced the antibacterial performance of the membranes, and M2~M4 demonstrated an improved antibacterial ability against *E. coli* and *S. aureus* to a certain extent, while also exhibiting a better dye CR separation ability (>67%). It is worth mentioning that M4 showed strong bacteriostatic action against both bacteria. The antibacterial activity of several materials was quantitatively evaluated using a CFU counting method. It was concluded that the bacteriostatic rate of M4 against *E. coli* was 50%, twice that of the pure MXene membrane. The inhibition rate of M4 against *S. aureus* was 82.4%, while that of pure MXene was only 31.7%. In this work, MXene-based 2D–2D composite membrane materials were constructed to improve the separation and antibacterial properties of membranes, providing a new idea for alleviating the biological contamination of membranes.

## Figures and Tables

**Table 1:** The detailed composite of different two-dimensional membranes.

Membrane	MXene (mg)	GO (mg)	O-g-C_3_N_4_ (mg)	BiOCl (mg)
M1	2	0	0	0
M2	2	1	0	0
M3	2	0	1	0
M4	2	0	0	1

## Data Availability

The data that support the findings of this study are available.
